# Trustworthiness of Web-Based Pharmacy Apps in Pakistan Based on the Mobile App Rating Scale: Content Analysis and Quality Evaluation

**DOI:** 10.2196/59884

**Published:** 2025-04-17

**Authors:** Anum Sattar, Hina Rehman, Safila Naveed, Sumaira Khadim, Nargis Khan, Ahmad Furqan Kazi, Wajid Syed, Mahmood Basil A Al-Rawi, Shazia Jamshed

**Affiliations:** 1 Department of Pharmacy Practice, Jinnah University for Women, 5C, Block 5 Nazimabad, Karachi, 74600, Pakistan, 92 3443786840; 2 Department of Pharmacy Practice, Faculty of Pharmacy and Pharmaceutical Sciences, Ziauddin University, Karachi, Pakistan; 3 Department of Pharmacy Practice, Institute of Pharmaceutical Sciences, Jinnah Sindh Medical University, Karachi, Pakistan; 4 Department of Pharmaceutical Chemistry, University of Karachi, Karachi, Pakistan; 5 Department of Pharmacy Practice, Faculty of Pharmacy, Iqra University, Karachi, Pakistan; 6 Department of General Medicine, Dow University of Health Sciences, Karachi, Pakistan; 7 Department of Pharmacy Practice, College of Pharmacy, Purdue University in Indianapolis, Indianapolis, United States; 8 Department of Clinical Pharmacy, College of Pharmacy, King Saud University, Riyadh, Saudi Arabia; 9 Department of Optometry College of Applied Medical Sciences, King Saud University, Riyadh, Saudi Arabia; 10 Department of Pharmacy Practice, School of Pharmacy, International Medical University, Kuala Lumpur, Malaysia

**Keywords:** online pharmacy, online pharmacy apps, apps, mobile app, smartphone app, trustworthiness, pharmacy, digital platforms, questionnaire, engagement, functionality, Mobile App Rating Scale, MARS, user, efficacy, Pakistan

## Abstract

**Background:**

Web-based pharmacy apps facilitate the electronic exchange of health-related supplies. They are digital platforms that run on websites and smartphones. Pakistan is experiencing significant progress in smartphone integration and digital services, leading to the expansion of the online pharmacy business. However, concerns remain over the legitimacy and precision of these apps.

**Objective:**

The aim of this study was to undertake a thorough assessment of digital pharmacy apps accessible in Pakistan. Specifically, our focus was on apps accessible via the Google Play Store and the iOS App Store. To fulfill this objective, an evaluation of these apps was performed using the Mobile App Rating Scale (MARS).

**Methods:**

A research investigation was conducted to analyze the online pharmacy apps in Pakistan. Initially, 50 apps were identified, but 10 were excluded for not meeting pre-established criteria, 10 were excluded for being in languages other than English, and 7 could not be downloaded. All paid and non-English apps were also excluded. A total of 23 apps were selected for the study, acquired via the Google Play Store and iOS App Store. The evaluation was conducted by 2 researchers who maintained independence from one another by using the MARS.

**Results:**

Initially, 50 apps were identified, of which 27 were excluded for not meeting the predetermined criteria. A total of 23 apps were selected for the study, acquired via the Google Play Store and iOS App Store. Strong positive correlations between higher user engagement and better app functionality and information quality were observed. The average rating of the 23 apps ranged between 2.64 and 4.00 on a scale up to 5. The aesthetics dimension had the highest mean score of 3.6, while the information dimension had the lowest mean score of 3.2. For credibility and reliability, different tests (intraclass correlation, Cohen κ, Krippendorff α, and Cronbach α) on each dimension of the MARS were performed by using SPSS Statistics 27. The intraclass correlation of all MARS dimensions ranged from 0.702‐0.913 (95% CI 0.521‐0.943), the Cohen κ of all MARS dimensions ranged from 0.388‐0.907 (95% CI 0.151‐0.994), the Krippendorff α of all MARS dimensions ranged from 0.705‐0.979 (95% CI 0.657‐0.923), and Cronbach α had a lower score of 0.821 in the information dimension and a higher score of .911 in the subjective quality dimension of the MARS.

**Conclusion:**

This study evaluated online pharmacy apps in Pakistan by using the MARS. It is the first study on online pharmacy apps in Pakistan. The findings of the evaluation have provided insights into the reliability and efficacy of these apps.

## Introduction

### Background

Over the past decade, the internet has evolved into a primary source of knowledge for many individuals. The demand for purchasing things online, including medications, has experienced a parallel surge [1]. In the last few years, a discernible transformation has occurred within the pharmacy industry, whereby a considerable number of conventional pharmacies have adopted e-commerce platforms. These internet-based pharmacy firms engage in commercial activities through web-based platforms, enabling the immediate distribution of drugs and health care equipment to clients using postal services.

The field of online pharmacies has seen significant market value growth in the last few years. In the United States, online pharmacies have raised about $29.35 billion in 2014 and are projected to reach US $128 billion by 2023, reflecting a yearly increase of 17.7% [2]. The main factors influencing the customer preference for online pharmacies are convenience, affordability, and availability of drugs that may not normally be accessible or may be subject to cancellation [3,4]. The spread of mobile and wireless gadgets on a worldwide scale has generated an exceptional and incomparable potential for the provision of global medical care. According to data provided by the International Telecommunication Union, the global number of digital phone connections reached over 6 billion in 2012, representing an 86% market share globally. It is noteworthy that more than 70% of these customers were located in economically deprived nations such as Pakistan [5]. However, a significant number of customers residing in developing nations have shown reluctance toward accessing pharmacies on the internet. The reluctance to use such networks might be linked to multiple factors, which include a lack of awareness of their functionality, concerns regarding their reliability and authenticity, and limited accessibility. The main catalyst for the widespread use of e-pharmacies by most clients is expediency [6]. A survey reveals that over 80% of persons living in disadvantaged areas have use of mobile telephone connections. The geographical reach of these networks encompasses approximately 90% of the globe’s population [7]. Internet pharmacies are rapidly growing in Pakistan. Organizations want to build trust and establish a favorable bond with customers. Pakistan’s internet-based pharmacy business is expected to develop at a compound annual growth rate of 16.8% between 2020 and 2025 [8]. The growth might be ascribed to a significant shift in customer preferences, as they progressively favor the use of such services. An extensive examination is necessary to investigate the current condition and incidence of online shopping for medicines in Pakistan, including established sites like Dawaai.pk, DVAGO, and Tabiyat.pk, among others. The predominantly digital nature of web-based pharmacies, except for the combination model of DVAGO, has significantly influenced client preferences [9]. The industry ensures medicine legitimacy and follows regulations [10]. The use of health care apps and electronic gadgets is crucial for both persons who are using them for self-management and clinicians who are using them to enhance health care, as they have the ability to access metrics that allow them to evaluate the effectiveness and security of these innovations [11]. Potential risks to people’s safety and well-being may arise if health-related apps and gadgets are not subjected to thorough assessments of their suitability and credibility. The lack of reliability in corroborating information inside medical apps has been emphasized in multiple studies. The increasing concern over the possible risks linked to the use of medical apps has led to an upsurge in curiosity in the surveillance of the effectiveness and dependability of these apps. Although there are no regulations in Pakistan regarding the usage of mobile health (mHealth) apps, we used international guidelines as a reference. In 2013, the Food and Drug Administration issued a handbook with guidelines for evaluating the quality of mobile health apps. Another guideline on digital health was established by the World Health Organization in order to enhance patient outcomes and provide direction for the application of initiatives related to digital health [12,13]. In the last decade, a research team led by Stoyanov developed the Mobile App Rating Scale (MARS) [14]. The MARS constitutes a well-recognized, dependable evaluation instrument designed exclusively for the assessment of the capabilities of (mHealth) apps [14,15]. The method has repeatedly shown a high level of dependability and a broad range of applications after being used for comprehensive evaluation of smartphone app content. The MARS is composed of 23 discrete elements that are organized into several areas, including participation, utility, design, accuracy of data, and subjective assessment [15-26].

### Study Objective

The aim of our research was to evaluate digital pharmacy apps in Pakistan that were available on the Google Play Store and the iOS App Store. To achieve the aforementioned goal, an evaluation of these apps was conducted using the MARS.

## Methods

### Ethical Considerations

The investigation was done with scrupulous attention to ethical guidelines. The investigators acquired ethical permission from the Institutional Ethics Review Board committee of Jinnah University for Women. Their authorization was granted under the reference number JUW/IERB/PHARM-ERA-004/2023. This measure was used to enhance the dependability and credibility of the study while upholding the highest ethical standards.

### Details of Assets and Investigation Techniques

A pair of assessors (pharmacists) was selected to evaluate the online pharmacy apps. The inclusion criteria required both assessors to have expertise in pharmacy and training in the MARS. The exclusion criterion was to eliminate individuals who were not trained in the MARS. Both assessors searched for web-based pharmacy apps on the official marketplaces of the 2 prominent operating systems, namely Android (Play Store) and iOS (App Store). To guarantee the comprehensiveness of the findings, a widely searched phrase, namely “online pharmacy apps in Pakistan,” was used in both stores.

### App Criteria for Acceptability

The study’s sample selection criteria were used to determine which apps met the specific inclusion criteria and exclude those apps that did not meet the inclusion criteria. Inclusion criteria included apps solely originating from Pakistan, accessible from the Google Play Store and iOS App Store, and which involved online pharmacies. Financial or premium app-related biases were not taken into account, and only English-language apps were considered. Exclusion criteria encompassed the exclusion of paid apps, apps in languages other than English, apps not available for download on the Google Play Store and iOS App Store, and any duplicate apps.

### The Attributes and Qualities Associated With the Apps

The complete dataset of the apps included the input of crucial information. The collection of data encompassed several attributes of the apps, such as their title, platform (Android or iOS), costs, class (about health care, wellness, and physical activity), date of the latest update, the newest language, number of reviewers, score, creator, and number of downloads.

### Selection Process of Web-Based Apps

The 2 neutral assessors assessed the apps by name and downloaded them from the iOS App Store and Google Play Store. The chosen apps demonstrated probable suitability and were entered into a database. The apps that satisfied the established criteria were kept, but those that failed to meet the requirements were eliminated. In instances when uncertainty arose over the suitability of an app, a third assessor was engaged.

### Gathering and Assessment of Information Integrity

The 2 assessors received instructions regarding the use of the MARS via individually viewing tutorial videos accessible on YouTube. Subsequently, the users proceeded to independently download, use, and assess the quality of apps. To gather the data, the researchers used a collection form that encompassed various details such as the app’s creator, device, version, release year of the latest release, costs, the number of downloaded files, feedback from consumers, the existence of a statement of confidentiality, privacy-related technical factors, medical device data, and the elements evaluated through the MARS.

### Investigational Tool

The assessment of the usability of the incorporated apps was carried out by using the MARS, a comprehensive tool including 23 criteria that are categorized into 5 distinct sections.

The engagement component of the assessment evaluates many factors including enjoyment, interest among users, personalization, interaction (including alerts, messages, signals, and comments), and suitability for the target demographic.

The functionality component encompasses 4 key aspects related to operational efficiency, including (1) utilization, (2) directions, (3) conceptual flow, and (4) gestural design. The part on aesthetics assesses the elements of graphic layout, visual attractiveness, chromatic palette, and aesthetic coherence. The next part examines the existence of quality data such as textual content, comments, evaluations, and citations derived from trustworthy sources.

The subjective quality component consists of 4 items. This section assesses the consumer’s level of enthusiasm for the app. Every single aspect was assessed using a rating system ranging from 1 (representing inadequacy) to 5 (representing excellence). The composite app grade was determined by computing the average score across parts A, B, C, and D. The app’s performance results are a subjective measure, which was derived separately by calculating the mean value of subsection E. Furthermore, subsection F included 6 app-specific questions that evaluated the perceived influence of the app on the user’s understanding, mindset, intention to make a change, and the probability of achieving successful modification of behavior concerning the targeted wellness behavior. To improve the objectivity of the MARS in evaluating app excellence, the inclusion of the subjective aspect of the quality part was omitted from the computation of the general average app performance grade. Moreover, the robust association shown in prior research between the MARS sum score and the individual’s star rating suggests its efficacy in reflecting the overall perceived excellence [14].

### Data Compilation and Assessment

Each reviewer conducted an independent evaluation of all the elements under the criteria of MARS for each of the apps. The average rating for each item within each app was computed using the numbers supplied by both evaluators. The mean scores for every category and part of the MARS were computed by taking the average values obtained from all the apps. As a result, mean scores were calculated for each category, and the same method was used to get the overall score and its corresponding standard deviation for each app.

### Statistical Evaluation

The mean score for each dimension of the MARS was determined by calculating the mean values as recommended by the raters. In addition, the mean and standard deviation were obtained and Pearson correlation analysis was conducted for all dimensions of the MARS. The interrater reliability estimate for the MARS tool was conducted using 3 statistical indicators (Cohen κ coefficient, Krippendorff α, and intraclass correlation coefficient [ICC]). The κ coefficient was evaluated by assigning quadratic weights to different values. The ICC was calculated using a 2-way random model to assess the degree of agreement. Weighted κ, Krippendorff α, and ICC were calculated for each dimension.

## Results

### Overview

**Figure 1.**
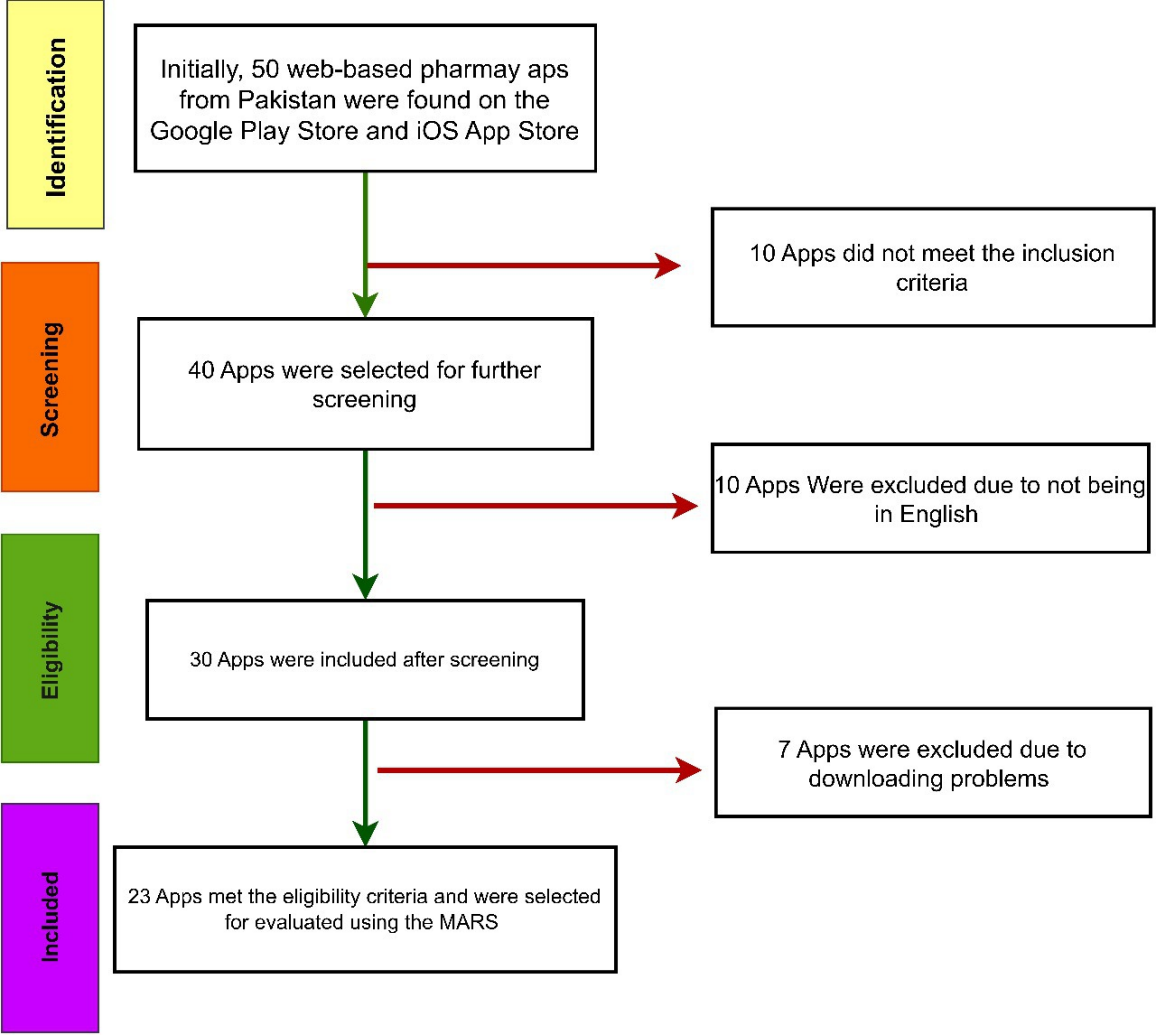
Flowchart for the selection of apps for this study.

A preliminary search was conducted on the Google Play Store and iOS App Store to identify 50 online pharmacy apps accessible in Pakistan, as demonstrated in Figure 1. A total of 10 apps were eliminated from the study due to their failure to meet the predetermined inclusion criteria. Additionally, 10 apps were found to be in languages other than English, so they were not included in the analysis. Furthermore, 7 apps could not be downloaded. A total of 23 apps were chosen for inclusion in the study, as shown in Figure 1. The apps have been downloaded from the Google Play Store and the iOS App Store, and they were evaluated using the MARS.

### App Characteristics

Web-based pharmacy apps in Pakistan offer a wide range of health care and pharmaceutical products and services. The tables in this section provide detailed information on technical features, creator details, customer reviews, pricing, version specifications, affiliations, and other relevant details of apps. These tables can aid users in selecting suitable apps and provide valuable insights into Pakistani health care and pharmaceutical competition. Table 1 presents a complete overview of the technological attributes associated with various pharmaceutical apps. Every app has been assigned a distinct serial number that uniquely identifies it and this table provides a comprehensive overview of the apps’ special technological capabilities. These qualities encompass the ability to distribute material across multiple platforms and offer secure login capabilities, among other functionalities. The table is presented as a valuable resource for both customers and developers, facilitating their comprehension of the various functionalities and fundamental potentials of these apps. Table 2 presents a summary of the main domains of focus (ie, strategies for utilization and the affiliations with which they are connected). The analysis suggests that the major objective of these kinds of apps is to enhance the welfare of individuals, while concurrently implementing a business-oriented approach that involves severing connections between companies. This information facilitates users’ understanding of the underlying goals and target market of these apps. Table 3 provides a comprehensive analysis of the manufacturers of pharmacy apps, encompassing essential details such as the number of evaluations, feedback, the date of the most recent update, and the pricing model of the app (whether it is free or paid). The table presented herein can be used by users to evaluate the reliability of these programs by taking into account both the reputation of the creators and the feedback offered by consumers. Table 4 presents a comprehensive examination of several versions of pharmaceutical software including their download statistics, release dates, and the operating systems on which they may be accessed. The provided data hold significant value for users who are in search of compatibility with their devices and the most up-to-date versions of apps. Table 5 presents concise and informative descriptions of pharmacy apps, furnishing users with a concise summary of the features and distinctive value proposition of each app. This feature is especially advantageous for individuals who are in search of a suitable app that meets their health care and pharmaceutical needs.

**Table 1.** Technological components of mobile apps for online pharmacies in Pakistan.

Serial number	App name	Technical aspects of the app (all that apply)
1	DVAGO	Allow sharing of different platforms, send reminders
2	Dawaai - Medicine & Healthcare	Allow sharing of different platforms, login protection
3	Servaid plus	Allow sharing of different platforms
4	Ehad Pharmacy	Allow sharing of different platforms, login protection
5	Goli Monthly Pharmacy	Allow sharing of different platforms, login protection
6	Dawawala	Allow sharing of different platforms
7	Sakoon Pharmacy and Healthcare	Allow sharing of different platforms
8	Emeds Pharmacy	Allow sharing of different platforms
9	Najeeb pharmacy	Allow sharing of different platforms
10	Tabiyat.pk	Allow sharing of different platforms
11	DawaAppTak	Allow sharing of different platforms, login protection
12	e-Clinix	Allow sharing of different platforms
13	Medicalstore.com.pk	Allow sharing of different platforms
14	Dava Pakistan	Allow sharing of different platforms
15	Qarshi	Allow sharing of different platforms
16	Medipanda	Allow sharing of different platforms
17	DH Pharmacy	Allow sharing of different platforms
18	Global Care	Allow sharing of different platform
19	Dawa Asaan	Allow sharing of different platforms
20	Bin Hashim	Allow sharing of different platforms
21	Dawa Online	Allow sharing of different platforms, login protection
22	Caplet	Allow sharing of different platforms
23	Care pharmacy	Allow sharing of different platforms, login protection

**Table 2.** Qualities and targets of online pharmacy apps in Pakistan.

Serial number	App names	Focus (what the app targets)	Theoretical background and strategies	Affiliations
1	DVAGO	Well-being	Business	Commercial
2	Dawaai - Medicine & Healthcare	Well-being	Business	Commercial
3	Servaid plus	Well-being	Business	Commercial
4	Ehad	Well-being	Business	Commercial
5	Goli monthly pharmacy	Well-being	Business	Commercial
6	Dawawala	Well-being	Business	Commercial
7	Sakoon pharmacy and health care	Well-being	Business	Commercial
8	Emeds	Well-being	Business	Commercial
9	Najeeb pharmacy	Well-being	Business	Commercial
10	Tabiyat.pk	Well-being	Business	Commercial
11	DawaAppTak	Well-being	Business	Commercial
12	e-Clinix	Well-being	Business	Commercial
13	Medicalstore.com.pk	Well-being	Business	Commercial
14	Dava Pakistan	Well-being	Business	Commercial
15	Qarshi	Well-being	Business	Commercial
16	Medipanda	Well-being	Business	Commercial
17	DH Pharmacy	Well-being	Business	Commercial
18	Global Care	Well-being	Business	Commercial
19	Dawa Asaan	Well-being	Business	Commercial
20	Bin Hashim	Well-being	Business	Commercial
21	Dawa Online	Well-being	Business	Commercial
22	Caplet	Well-being	Business	Commercial
23	Care pharmacy	Well-being	Business	Commercial

**Table 3.** Information about the creator and specific characteristics of online pharmacy apps in Pakistan.

Serial number	App name	Developers	Number of reviews	Rating	Last update	Cost
1	DVAGO	Novacare (Pvt) Ltd	725	3.5	July 3, 2023	Free
2	Dawaai - Medicine & Healthcare	Dawaai Pvt Ltd	12,600	3.6	April 21, 2023	Free
3	Servaid plus	Servaid Pharmacy	461	2.4	July 19, 2023	Free
4	Ehad	Ehad Virtual Healthcare	72	3.9	December 12, 2022	Free
5	Goli monthly pharmacy	Atlash Tech LLC	85	4.5	January 31, 2023	Free
6	Dawawala	Dawawala	1000	4.9	April 29, 2022	Free
7	Sakoon pharmacy and health care	Healthcare Mart Pvt Ltd	33	4.5	August 25, 2023	Free
8	Emeds	Emeds	10	3.7	March 15, 2021	Free
9	Najeeb pharmacy	Xperia Tech	630	2.8	September 27, 2022	Free
10	Tabiyat.pk	Medznmore	4000	1.9	April 19, 2023	Free
11	DawaApptak	Muller & Phipps (Pvt.) Ltd	6	5	September 8, 2023	Free
12	e-Clinix	Bright-line solutions	195	3.2	April 5, 2022	Free
13	Medicalstore.com.pk	Medicalstore.com.pk	746	2.3	N/A^a^	Free
14	Dava Pakistan	B techno Media	16	3.9	November 25, 2021	Free
15	Qarshi	Obrotu	112	3.8	February 28, 2021	Free
16	Medipanda	Medipanda Pvt Ltd	42	3.8	November 24, 2020	Free
17	DH Pharmacy	Digital Health Services	0	N/A	August 30, 2023	Free
18	Global Care	Devcon Digital	10	4.0	July 20, 2017	Free
19	Dawa Asaan	Dawa Asaan	29	4.5	July 31, 2023	Free
20	Bin Hashim	Blink Co.Technologies	151	4.6	June 10, 2023	Free
21	Dawa Online	Dawa online	56	2	April 12, 2023	Free
22	Caplet	Salman abid	0	N/A	June 27, 2023	Free
23	Care pharmacy	Sibyl technologies	0	N/A	May 4, 2023	Free

a N/A: not applicable.

**Table 4.** Information about the apps, including details regarding their version updates, download statistics, platform compatibility, and associated age group.

Serial number	App name	Version	Downloads	Release date	Platform	Age group
1	DVAGO	2.1.5	≥100,000	January 31, 2022	iOS	General
2	Dawaai - Medicine & Healthcare	4.2.1	≥1,000,000	December 30, 2017	Google Play Store	General
3	Servaid Plus	1.2.2	≥50,000	December 4, 2020	iOS	General
4	Ehad	1.2.4	≥10,000	October 1, 2021	Google Play Store	General
5	Goli Monthly Pharmacy	1.0.1.0	≥10,000	October 31, 2022	iOS	General
6	Dawawala	1.0.9	≥1000	April 14, 2022	Google Play Store	General
7	Sakoon Pharmacy and Healthcare	1.0.2	≥1000	November 8, 2022	iOS	General
8	Emeds	1.0	≥1000	March 15, 2021	Google Play Store	General
9	Najeeb Pharmacy	31.1	≥10,000	August 15, 2020	iOS	General
10	Tabiyat.pk	4.1.1	≥100,000	April 16, 2021	Google Play Store	General
11	DawaAppTak	8.0.0	≥500	August 13, 2022	Google Play Store	General
12	e-Clinix	4.4	≥10,000	July 10, 2021	iOS	General
13	Medicalstore.com.pk	N/A^a^	100,000	N/A	Google Play Store	General
14	Dava Pakistan	0.0.1	≥1000	March 31, 2021	Google Play Store	General
15	Qarshi	2.0.6	≥10,000	April 22, 2020	Google Play Store	General
16	Medipanda	1.18	1000	November 9, 2019	Google Play Store	General
17	DH Pharmacy	1.01	≥10	April 21, 2022	Google Play Store	General
18	Global Care	1.1.4	≥100	July 11, 2017	Google Play Store	General
19	Dawa Asaan	3.1.0	≥1000	February 10, 2021	iOS	General
20	Bin Hashim	1.1.2	≥10,000	April 7, 2022	iOS	General
21	Dawa Online	1.0.4	≥10,000	January 31, 2020	Google Play Store	General
22	Caplet	2.01	≥50	April 7, 2022	iOS	General
23	Care pharmacy	1.01	≥1000	May 4, 2023	Google Play Store	General

a N/A: not applicable.

**Table 5.** Summary of online pharmacy apps in Pakistan.

Serial number	App name	Description provided by the app
1	DVAGO	DVAGO is Pakistan‘s most trusted pharmacy chain for complete health care
2	Dawaai - Medicine & Healthcare	Dawaai Pakistan, also known as Dawaai.pk, is Pakistan’s premier online pharmacy, offering a comprehensive 360-degree medical solution for all your health care requirements. Dawaai holds the distinction of being one of Pakistan’s longest-established and most reliable digital health platforms, providing access to affordable medications.
3	Servaid plus	Your medicine is at your doorstep, with Servaid, no more waiting in lines anymore.
4	Ehad	A better world with quality health care.
5	Goli monthly pharmacy	Goli is an online pharmacy platform.
6	Dawawala	B2B marketplace for pharmaceutical distributors and wholesalers.
7	Sakoon pharmacy and health care	A pharmacy of branded medicines in your pocket.
8	Emeds	Pakistan’s number 1 online pharmacy for online medicines, lab tests, and health tips.
9	Najeeb pharmacy	We provide free home delivery service in Islamabad within an hour.
10	Tabiyat.pk	Tabiyat.pk is an online pharmacy app where your medicines and health care products get delivered anywhere in Pakistan.
11	DawaApptak	DawaApptak customer app for our valuable customers.
12	e-Clinix	e-Clinix, the leading retail pharmacy brand of Pakistan, is now at your door.
13	Medicalstore.com.pk	The biggest online pharmacy app and medical store offers medicine delivery services in Pakistan.
14	Dava Pakistan	An online directory that is in the process of transitioning into an online pharmacy. Our goal is to offer people access to information about alternative medicines, helping them find cost-effective options rather than paying exorbitant prices.
15	Qarshi	Authentic health products with the fastest delivery.
16	Medipand	Get your medicines delivered right to your doorstep with convenient cash payment upon delivery. We also provide herbal deliveries, surgical machines, patient-use devices, wheelchairs, baby essentials, and food supplements, all available with free delivery and the option to pay cash on delivery. As Pakistan’s first trusted online pharmacy, we are your go-to source for purchasing medicines online in Lahore, Punjab, Pakistan.
17	DH Pharmacy	Pakistan‘s number 1 pharmacy for medicines, beauty, and surgical products.
18	Global Care	Global Care stands as Pakistan’s pioneering online pharmacy, offering the option to purchase affordable medicines through the convenience of uploading your doctor’s prescription or placing an order independently. We are a licensed pharmacy providing home delivery services, with delivery times of 1 to 2 hours in Rawalpindi - Islamabad and 2-3 days across Pakistan.
19	Dawa Asaan	Dawa Asaan is Pakistan’s first smart pharmacy.
20	Bin Hashim	Order your medications and various items online, and have them conveniently delivered to your doorstep.
21	Dawa Online	Dawa Online Medical Store is a prominent and recognized medical establishment situated in the city of Raipur. Their services encompass the timely delivery of medicines, over-the-counter products, cosmetics, and generic medications to cater to the needs of all their patients.
22	Caplet	The Caplet Pharmacy app facilitates a swift sign-up procedure and efficiently locates the medicines prescribed to you by your doctor. Additionally, Caplet Pharmacy extends its services to provide over-the-counter products to its customers.
23	Care pharmacy	Online marketplace for acquiring prescription medications and health-related items.

### MARS Assessment

**Figure 2.**
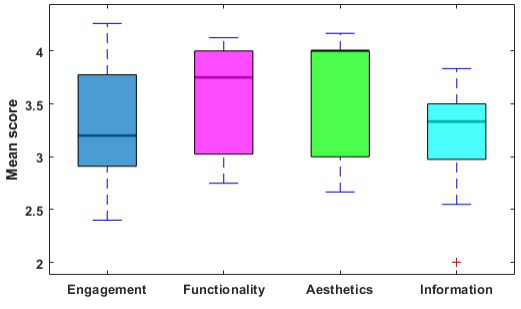
Mean scores of each dimension of the Mobile App Rating Scale.

**Figure 3.**
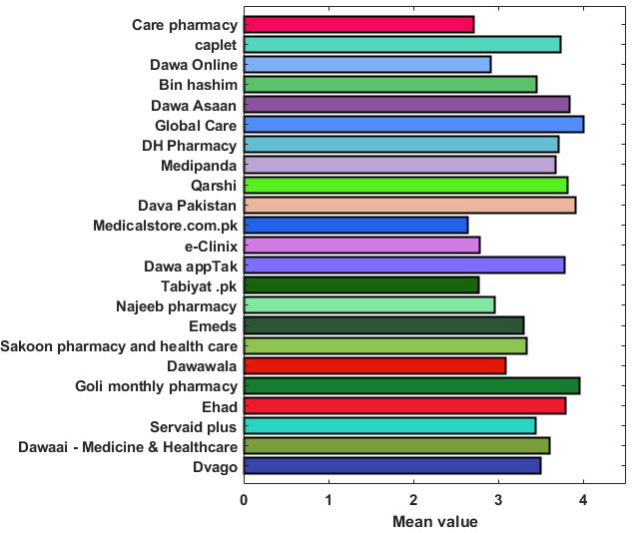
Mean scores of individual apps as evaluated by the Mobile App Rating Scale.

The mean scores of the 23 apps ranged from 2.64 to 4.00. The mean scores of each dimension of the MARS are demonstrated in Figure 2. The individual scores of each app are presented in Figure 3.

The ICC coefficient was computed for the 5 aspects of the MARS (Table 6). The ICC coefficient for the variable “Engagement” was determined to be 0.764, with a 95% CI ranging from 0.521 to 0.893. This finding indicates a considerable degree of consensus among raters about the concept of participation. Similarly, the construct labeled “Functionality” demonstrated a notable ICC coefficient of 0.824 (95% CI 0.630-0.922), which signifies a robust consensus among raters on their evaluations of the construct’s functionality. The variable “Aesthetics” demonstrated the highest ICC coefficient, measuring 0.949 (95% CI 0.885-0.978), indicating a substantial level of consensus about aesthetic judgments. The dimension of “Information” demonstrated a moderate ICC cofficient of 0.702 (95% CI 0.601-0.875), and “Subjective quality” exhibited strong agreement, as indicated by an ICC coefficient of 0.871 (95% CI 0.720-0.943). The Krippendorff α coefficient was computed to evaluate the reliability of the MARS over the identical set of 5 dimensions. The Krippendorff α coefficients for the dimensions of “Engagement,” “Functionality,” “Aesthetics,” “Information,” and “Subjective quality” were 0.759 (95% CI 0.661-0.857), 0.825 (95% CI 0.727-0.923), 0.979 (95% CI 0.881-1.077), 0.705 (95% CI 0.689-0.885), and 0.755 (95% CI 0.657-0.853), respectively. The Krippendorff α values, accompanied by their corresponding CIs, serve to affirm the reliability and coherence of raters’ interpretations across the assessed factors, so enhancing the overall comprehension of the data’s integrity. Furthermore, the investigators used weighted κ statistics to evaluate the characteristics of the MARS. The weighted κ value for the parameter “Engagement” was calculated to be 0.764 (95% CI 0.350-0.722), indicating a reasonable degree of consistency that has been adjusted for chance. The assessment of “Functionality” resulted in a Cohen κ score of 0.658 (95% CI 0.510-0.805), showing a statistically significant degree of consensus among evaluators about the meaning of functionality. The variable “Aesthetics” demonstrated a statistically significant κ value of 0.907 (95% CI 0.819-0.994), showing a strong agreement in the evaluation of aesthetic attributes. The κ statistic for the value of the parameter “Information” was calculated to be 0.388 (95% CI 0.151-0.626). The study’s findings indicate a rather low degree of consensus between assessors about the dimension of Information. The subjective quality assessment resulted in a κ score of 0.670 (95% CI 0.488-0.851), showing a statistically significant level of agreement. The reliability and internal consistency of every element of the MARS questionnaire were assessed using Cronbach α. The rating of “Engagement” had a Cronbach α value of 0.867, indicating a high degree of internal consistency. The concept of “Functionality” demonstrated a significant degree of internal consistency, as indicated via a Cronbach α value of 0.904. The concept of “Aesthetics” had a notably high Cronbach α rating of 0.905, suggesting a commendable level of internal consistency. The Cronbach α value of 0.821 indicates that the “Information” parameter has a high level of internal coherence. The construct of “Subjective quality” demonstrated a Cronbach α coefficient of 0.911, indicating a substantial degree of internal consistency.

**Table 6.** Evaluating the consistency and reliability of online pharmacy apps in Pakistan using the intraclass correlation coefficient, Krippendorff α, κ, and Cronbach α.

	Values		
**Mobile App Rating Scale dimensions, intraclass correlation coefficient (95% CI)**			
Engagement	0.764 (0.521-0.893)		
Functionality	0.824 (0.630-0.922)		
Aesthetics	0.913 (0.885-0.978)		
Information	0.702 (0.601-0.875)		
Subjective quality	0.871 (0.720-0.943)		
**Mobile App Rating Scale dimensions, Krippendorff α (95% CI)**			
Engagement	0.759 (0.661-0.857)		
Functionality	0.825 (0.727-0.923)		
Aesthetics	0.979 (0.881-0.077)		
Information	0.705 (0.689-0.885)		
Subjective quality	0.755 (0.657-0.853)		
**Mobile App Rating Scale dimensions, weighted κ (95% CI)**			
Engagement	0.536 (0.350-0.722)		
Functionality	0.658 (0.510-0.805)		
Aesthetics	0.907 (0.819-0.994)		
Information	0.388 (0.151-0.626)		
Subjective quality	0.670 (0.488-0.851)		
**Mobile App Rating Scale dimensions, Cronbach α**			
Engagement	0.867		
Functionality	0.904		
Aesthetics	0.905		
Information	0.821		
Subjective quality	0.911		

### Relationship of MARS Dimension With Total MARS Scores and the Mean (SD) of Each Dimension

Table 7 presents a detailed overview of the relationship values, means, and standard deviations of various elements of the MARS. The dimension denoted as “Engagement” exhibited a notable favorable correlation of 0.949. The average score for this aspect was 3.324, accompanied by a rather small standard deviation of 0.560. The results of this study indicate a substantial degree of concurrence and uniformity among participants in their assessments of engagement. The parameter “Functionality” demonstrated a significant positive relationship of 0.923. The data exhibited a marginally elevated mean rating of 3.520, accompanied by an acceptable standard deviation of 0.491. These findings indicate consistent perceptions of the functionality of the subject matter. The aspect referred to as “Aesthetic” demonstrated a considerable positive association of 0.798. Additionally, it had a mean value of 3.626 and a standard deviation of 0.504. The aforementioned figures indicate a substantial agreement among participants on their assessments of beauty. The aspect labeled as “Information” exhibited a noteworthy positive relationship of 0.763. The average grade for this aspect was 3.044, suggesting a consistent perspective. Moreover, the observed standard deviation of 0.359 indicates a rather small range of variation in individuals’ judgments regarding the information aspect. The study’s findings provide valuable insights into the prevailing viewpoints of the participants and the level of agreement seen across multiple aspects. These observations enhance our understanding of the information quality derived from the MARS.

**Table 7.** Mean and standard deviation of the correlation among various aspects of the Mobile Rating App Scale.

Mobile App Rating Scale dimensions	Correlation value	Mean (SD)	
Engagement	0.949	3.324 (0.560)	
Functionality	0.923	3.520 (0.491)	
Aesthetic	0.798	3.626 (0.504)	
Information	0.763	3.044 (0.359)	

## Discussion

### Principal Findings

Online pharmacies not only offer medication selling, purchasing, and delivery to patients but they also provide medication details, which include brand, dosage form, indication, side effects, warning, and price. Thus, the critical first step in the treatment process is to evaluate the standards of online pharmacies. This investigation represents a significant achievement as it constitutes the inaugural evaluation of the quality of online pharmacy apps in Pakistan through the utilization of a mobile app grading scale developed by Stoyanov et al [14]. In this comprehensive study, a meticulous analysis was undertaken of the pharmacy apps available in Pakistan; as Amor-García et al [12] did with genitourinary tumor apps, we chose and downloaded our apps using both the Google Play Store and the iOS App Store. The sample size of 23 apps was comparable to the sample size in research carried out by Kim et al [26]. These apps met the established criteria. As previously reported in other research, we discovered that the Google Play Store had a wider variety of apps than the iOS App Store. Nine apps were downloaded from the iOS App Store, while 14 came from the Google Play Store [21,27].

The MARS is an innovative and effective tool that is used for evaluating mobile health apps. The mean score of all apps ranged from 2.64 to 3.92, which was similar to scores reported by previous studies [17,28-30]. The subjective quality component of the MARS was omitted from the collective mean score [24,31]. Furthermore, we determined the average scores for several dimensions of “Engagement” (mean 3.32, SD 0.560), “Functionality” (mean 3.52, SD 0.49), “Aesthetics” (mean 3.62, SD 0.50), and “Information quality” (mean 3.04, SD 0.35). Several methodologies have been developed to evaluate the efficacy of mHealth apps. Nevertheless, the absence of a universally accepted method and the limited participation of scientific professionals have made it impractical to regularly put them into practice [32]. The MARS approach, as documented by Stoyanov et al [14], is regarded as a user-friendly, straightforward, and coherent instrument that is highly regarded for its reliability due to its endorsement by skilled technicians and health care experts. This scale enables a comprehensive analysis that encompasses several disciplines and is intended for the assessment of health care apps. It consists of 5 domains that encompass the key factors necessary for a thorough evaluation. In their study, Stoyanov et al [14] found that the MARS assessment had excellent internal consistency (Cronbach α=0.9) and interrater reliability (2-way mixed ICC coefficient=0.79, 95% CI 0.75‐0.83) when used to assess 50 mental health apps.

Our findings were similar to both the original MARS and other translated versions, according to reliability and validity studies [33-36]. As per globally recognized quality standards, all sections’ internal reliability and Cronbach α as well as the overall scores were found to be good [37]. Subsequent translation studies and the original MARS study also noted this high level of internal consistency. In contrast, our research revealed a good degree of interrater reliability, with an ICC ranging from 0.702 to 0.913. To ensure construct validity, we opted for a multitrait scale evaluation with the item-subscale association over a factor analysis, as this approach has proven effective in prior research [26]. Our findings met the predetermined success level and demonstrated good parallel and divergent reliability, except for the “Information” subscale because many parameters were not measurable and less information on content related to medications. Nonetheless, we discovered a low ICC for the “Information” subscale. Prior research has similarly documented the diminished outcomes associated with the information variable [17].

The mobile apps provided did not consistently cite the authors or websites that provided information, and there was no assurance of the accuracy and timeliness of the material.

The diminished quality of data has been linked to a variety of dangers for mobile app users, primarily due to the potential for misinformation leading to inaccurate self-diagnoses and unfavorable health-related decisions in prevention, wellness, and therapy [38,39]. The majority of apps were simple to download and log-in friendly. Of the 23 apps, we only encountered login issues with 2 or 3. In contrast to other aspects of the MARS, the information dimension was reduced because, of the 23 apps, some apps provided information about the specifics of medications, such as dosage forms, indications, side effects, precautions, categories, and alternative brands, and many apps only presented brand names with the cost and category of drugs. The medication information is useful for patients to have in addition to the drugs they choose or for other purposes relevant to medicines or managing their treatment.

As a result of transportation and public gathering limitations brought on by the COVID-19 epidemic that struck the nation in February 2020, the selling of medicines online expanded significantly in the years that followed [40]. Many people started to order medications from online pharmacies even if the quality of the products was still questionable according to medical professionals. Pharmacists argued that online pharmacies put their businesses at risk and allow the free selling of potentially harmful drugs [41-46]. The actual number of online pharmacies in Pakistan is unknown, but according to authorities, most of them are located in the country’s 2 biggest cities, Karachi and Lahore. They claim that, in specific locations, clients will get prescription drugs and other medical supplies within 4 hours of placing their order, or, in other cities, within 48 hours via a shipping service. In addition, many online pharmacies provide free shipping to client residences and discounts of as much as 20%. Online pharmacies help people get medicines in just a few clicks. Online pharmacies are not only a source of medicine delivery. Online pharmacy apps provide other services including prescription refills, information about the availability of drugs, and information about drugs. Pharmacies are the main pillar for this treatment method [47,48]. However, there have been no studies performed on this topic. Our study provides information about these pharmacy services, including engagement, functions, layout, and basic information.

### Limitations

The scope of our research was mostly limited to a single geographic region, namely Pakistan, and specifically focused on apps accessible in the English language. Additionally, the limitations of our study stem from the narrow focus on free apps. We did not conduct a thorough literature analysis and our technique was primarily focused on app stores. As a result, it might be possible that we did not fully explore the entire collection of available data. The evaluation was done by just 2 assessors. There were more assessors in previous studies that used the MARS.

### Future Recommendations

To bolster the reliability of online pharmacy apps in Pakistan, it is advisable to enforce more stringent laws and guidelines, regularly assess the content of these apps through audits, fortify data security measures, and provide standardized and evidence-based medical information. Subsequent investigations may focus on the exploration of client observations and opinions about these apps, with a particular emphasis on the dynamics of user-platform interaction and the establishment of trustworthiness.

### Conclusion

The MARS is a trustworthy and adaptable tool for assessing the reliability of mobile apps. The evaluation of online pharmacy apps of Pakistan using the MARS provided helpful data on the reliability and efficacy of these apps. The results showed the trustworthiness and dependability of web-based pharmacy apps. Many of the evaluated online pharmacy apps exhibited high reliability and credibility in all dimensions of the MARS.

## Abbreviations

ICC: intraclass correlation
MARS: Mobile App Rating Scale
mHealth: mobile health
