# Correction: Trustworthiness of Web-Based Pharmacy Apps in Pakistan Based on the Mobile App Rating Scale: Content Analysis and Quality Evaluation

**DOI:** 10.2196/88648

**Published:** 2026-03-03

**Authors:** JMIR Publications Editorial Office

**Affiliations:** 1 JMIR Publications, 130 Queens Quay E, Suite 1100, Toronto, ON, M5A 0P6, Canada, +1 416 583 2040

Following the publication of “Trustworthiness of Web-Based Pharmacy Apps in Pakistan Based on the Mobile App Rating Scale: Content Analysis and Quality Evaluation” [1], concerns were raised regarding potential conflicts of interest between reviewers Huma Ali and Sadia Shakeel and the authors. These concerns included prior copublication with members of the author list and an active professional relationship between a reviewer and an author. These conflicts were not disclosed to the JMIR Publications Editorial Office as per journal policy.

This article has been subsequently reassessed by the editor in chief, who determined that the content of these reviews did not unduly influence the decision to accept the article. The decision to accept the submission was based primarily on the remaining reviewer’s comments and the editor in chief’s contemporary appraisal of the manuscript.

As such, the JMIR Publications Editorial Office issues this corrigendum to update the Conflicts of Interest statement:

Reviewers Huma Ali and Sadia Shakeel were found to have undisclosed conflicts of interest with members of the author list under the terms of JMIR Publications policy. The editor in chief determined that the content of the reviews did not unduly influence the decision to accept the article. The decision to accept the submission was based primarily on the remaining reviewer’s comments and the editor in chief’s contemporary appraisal of the manuscript.

We regret that these issues were not identified before publication.

The correction will appear in the online version of the paper on the JMIR Publications website together with the publication of this correction notice. Because this was made after submission to PubMed, PubMed Central, and other full-text repositories, the corrected article has also been resubmitted to those repositories.
